# Finding Silver Linings in the Covid-19 Pandemic: A 2-Wave Study in the UK

**DOI:** 10.1177/00332941231219788

**Published:** 2023-11-30

**Authors:** Alison M. Bacon, Jaysan Charlesford, Michael Hyland, Tilla Puskas, Poppy Hughes

**Affiliations:** School of Psychology, 6633 University of Plymouth, Plymouth, UK

**Keywords:** Well being, positive psychology, Covid-19, silver lining, adversarial growth, big five

## Abstract

The Covid-19 pandemic has resulted in widespread anxiety, fear and depression, yet focussing only on these negative issues may obscure the opportunity to promote positivity and resilience. Traumatic events can often result in positive life changes (adversarial growth) though there is little evidence in the context of pandemics, and no previous studies in Covid-19 with the general public. The present research investigated whether adversarial growth was perceived in Covid-19 and whether this could account for variance in wellbeing, over and above effects of personality traits. Participants recruited from the UK public (*N* = 183) completed the Big Five Personality Inventory, the WHO-5 Wellbeing Scale and the Silver Lining Questionnaire (SLQ) measure of adversarial growth. Questionnaires were completed online, at two timepoints, nine months apart. At Time 1, wellbeing was negatively associated with trait Neuroticism and positively associated with Openness to experience. Both associations were positively mediated by SLQ score. At Time 2, SLQ score again mediated the effects of Openness on wellbeing, and also the influence of wellbeing at Time 1 on that at Time 2. Reported Silver Linings included strengthened personal relationships at Time 1, and improved ability to handle life events at Time 2. This suggests a shift from an appreciation of relationships to an awareness of personal development once life returned to some semblance of normality. Overall, results suggest that perceived adversarial growth supported wellbeing during the pandemic and highlight a focus for therapeutic intervention.

## Introduction

There is substantial evidence to indicate that Coronavirus Disease 2019 (COVID-19) has severely impacted wellbeing, and this impact is not limited to those who developed the disease. The pandemic and measures to reduce transmission have resulted in significant levels of stress (Brooks et al., 2020; Rodrigues, et al., 2021; Taylor & Amundsen, 2020), fear (Ahorsu et al., 2020; Luo, et al., 2021), and depression and anxiety (Fancourt et al., 2021; Lee, 2020). Fears of infection and repeated lockdown conditions have resulted in frustration, boredom and loneliness, as well as worries about prospective financial and employment loss and interruptions to children’s education (Brooks et al., 2020; Van Bavel et al., 2020). Ongoing effects on the economy and employment, along with posttraumatic psychological effects, present a continued threat (Douglas et al., 2020; Taylor & Asmundson, 2020). However, focussing only on the negative sequelae of the pandemic may mean that we miss the opportunity to develop interventions that encourage positivity and personal growth and therefore offer hope. The present study examines whether some people have been able to discover positivity in the experience (find a silver lining) and whether this has supported wellbeing during Covid-19. If so, this may have implications for intervention to address negative mental health repercussions of the pandemic.

“Finding a silver lining” is an idiomatic expression in English, which refers to situations in which something positive arises from a negative circumstance. In the psychology literature, such outcomes reflect what is often termed adversarial or posttraumatic growth, positive psychological effects experienced as a result of engaging with highly challenging or traumatic life events (Tedeschi et al., 2018). In the present context, we refer to as adversarial growth, because the term posttraumatic suggests that the negative situation is ended, and the Covid pandemic was still continuing at the time of our research. Adversarial growth occurs as a natural process, rather than as a result of intentional action or therapy. Traumatic situations challenge our assumptions about ourselves and the world and this can lead to a reassessment of life, discovery of meaning in adverse events, and the reconstruction of our sense of self and our world (Joseph & Linley, 2008; Tedeschi & Calhoun, 2004). Adversarial growth has been observed following major events such as the US terrorist attacks on September 11, 2001, and bombings in Madrid in 2004 (Linley & Joseph, 2011; Steger et al., 2008), as well as in the context of crime (Brooks et al., 2016), violence and abuse (Haslam et al., 2021), bereavement (Calhoun et al., 2010), miscarriage (Krosch & Shakespeare-Finch, 2017) and a wide range of other contexts (Linley & Joseph, 2004 for review). Much research has focussed on health outcomes, including cancer (McBride et al., 2009), HIV (Drewes et al., 2021), and Chronic Fatgue Syndrome (Hyland et al., 2006). A review of three categories of trauma experiences, health, accident, and those releting to professions such as firefighters, suggested that around 53% of people report at least some degree of adversarial growth (Wu et al., 2019). The consistant finding is that perceving a silver lining in adversity results in better adjustment and can counter, or overcome, the impacts of trauma.

However, evidence of adversarial growth in the context of pandemics is limited. Following the first Severe Acute Respiratory Syndrome (SARS) pandemic, Lau et al. (2006), reported that over 60% of participants reported spending more time with friends and family and experiencing greater feelings of caring and closeness. They also found more time for personal care and developed a greater sense of gratitude for positive aspects of their lives. That is not to say these individuals did not experience anxieties and concerns about the disease, but they were able to transcend those to experience personal and social growth. Only two studies have concerned Covid-19, both with very specific popualtions. Chen et al. (2020) reported data from a study of nurses where, despite high levels of burnout and trauma responses, 33% showed evidence of adversarial growth. Stallard et al. (2021) examined the experiences of child carers in Portugal and the UK. Over 88% answered in the affirmative to a question which asked whether they believed any positives had arisen from the Covid-19 pandemic, with the most common examples being the development of closer and more meaningful relaionships, followed by the opportunity to reassess what was most important in life. These studies suggest potential for silver linings in Covid-19, but both focussed on participants from very specific caring occupations, which arguably have very specific experiences having worked at the front line of healthcare. There is currently no evdidence regarding general public experiences and the present study addresses this limitation.

We also address possible individual differences in perceptions of adversarial growth. Posttraumatic/adversarial growth theory suggests that personality is a major determinant of the predisposition to growth (Tedeschi et al., 1998), and personality is also one of the most significant factors in psychological outcomes of pandemics generally (Taylor, 2019). As such, the present study considered personality traits as potential predictors of growth in Covid-19. Arguably the most widely reported personality model is the Five-factor framework (Costa & McCrae, 2006) which defines five traits: Conscientiousness (self-controlled and goal-oriented); Agreeableness (trusting, helpful, warm-hearted); Neuroticism (predisposition to psychological distress, depression, maladaptive coping); Openness to experience (creative, amenable to new ideas); and Extraversion (sociable and outgoing). These Big Five traits are of interest to the present research given evidence that Neuroticism is negatively associated with adversarial growth while the other traits are positively associated (Linley & Joseph, 2004), with Extraversion and Openness suggested to have the strongest influence (Linley & Joseph, 2004; Sheikh, 2004; Tedeschi & Calhoun, 2004; Tedeschi et al., 2018).

Given the nature of trait Neuroticism, it unsurprising that it has been associated with high levels of depression, anxiety, stress and health anxiety in response to Covid-19 (Aschwanden et al., 2020; Kocjana et al., 2020; Kroencke, et al., 2020; Nikčević et al., 2020). There is less research on the remaining Big Five traits, though extraverts may respond poorly to lockdown restrictions because sociability is intrinsically linked to their personal wellbeing (Carvalho et al., 2020; Wijngaards et al., 2021). Conversely, Conscientiousness, Openness and Agreeableness are all associated with emotional resilience, and relate to lower anxiety during the pandemic (see Bacon et al., 2022 for review).

### The Present Study

In summary, adversarial growth can lead to lasting positive outcomes following challenging and traumatic events, and there is evidence that certain personality types are more or less likely to experience such growth. However, little is known about adversarial growth in pandemics, and this is the first study to examine this question in the context of Covid-19 with a general public sample. This research is important because the pandemic is not only a negative life event on an individual level, but also a global disaster for which significant and widespread social aftereffects are anticipated (Douglas et al., 2020). Recognising ways in which people can thrive psychologically in the face of a serious threat to population health and the accompanying social upheaval, and identifying the individual differences which underpin this ability, can help to support positive interventions to address post-pandemic sequalae.

To this end, we investigated psychological wellbeing, adversarial growth and personality traits in a heterogeneous sample of the UK public across two timepoints, nine months apart, during the Covid-19 pandemic. We predicted:

1. Significant direct effects of Neuroticism (negatively) and of the other Big Five traits (positively) on wellbeing.
2. Perceived adversarial growth would account for variance in wellbeing in addition to that explained by personality traits.
3. Adversarial growth would mediate the effects of personality on wellbeing, thereby reducing the negative effects of Neurotism, and strenthening positive effects of other traits.
4. The most frequently endorsed benefits would relate to reassessment of life priorities and values, and strengthening of close relationships.

## Methods

### Time 1

Data were collected between 10th and 15th November 2021. There were no longer lockdown restrictions in the UK at this time. Mask wearing and social distancing were still encouraged indoors but were not mandated. The vaccination programme had progressed well with 68% of the UK population fully vaccinated, and 17% having received a third booster shot (Gov.UK, 2021a). News of the new omicron variant emerged on 12th November, and the Government announced a heightened risk for the British public and health services, although it was 27th November before the first UK cases emerged and advice to the public remained unchanged, (GOV.UK, 2021b).

#### Participants

To achieve a medium effect size (Cohen, 1988), an á priori power analysis with G-Power v.3.1 suggested a sample of 172 was required. We recruited 200 UK general public participants through Prolific.ac.uk, an online survey recruitment platform which provides representative and reliable samples (Woods et al., 2015). Our inclusion criteria were that participants must be aged 18 years or over, UK residents and have lived in the UK for at least 5 years to ensure they had experienced time before and during the period of social restrictions. Seventeen participants presented an incomplete dataset and were removed from the sample, leaving 183 for analysis (55 male, 125 female, 1 other; Mean age = 35.27, SD = 14.52, range 18–81). Subjective Socio-economic status (SES) was assessed by the MacArthur Ladder Scale (Adler et al., 2000) which presents a hypothetical ladder with 10 rungs, where higher rungs represent individuals who have most money, education, and prestigious jobs. Participants are asked to indicate their perceived position on this ladder. The mean report was 5.04 (SD = 1.96, range 1–10). One hundred and thirty-seven participants (75%) reported that they or someone close to them had experienced Covid-19 infection. Participants reported home regions throughout the UK, though 64% were based in South or South-West England.

#### Procedures and Materials

The study was approved by the authors’ university school ethics committee. Data were collected by an online survey hosted on the Qualtrics survey platform. Participants completed the following measures:

*Silver Lining Questionnaire* (SLQ; Sodergren & Hyland, 2000; Sodergren et al., 2002). The 38-item SLQ is a scale of adversarial growth and the ability to derive positive meaning from negative events and experiences. The scale was developed originally for use with illness experiences, and in the present study we substituted the words “my illness” with “the pandemic”, for instance the item *My illness strengthened my relationship with others*, became *The pandemic strengthened my relationship with others*. Participants were asked to indicate the extent to which they agreed with items on a scale where 1 = strongly disagree, and 5 = strongly agree. A value of 1 was allocated to responses of ‘strongly agree’ and ‘agree’, and a value of 0 to all other responses. These were summed for a total score reflecting the number of positive reflections on the pandemic. The SLQ presented excellent reliability with the present sample (α = .94).

*The World Health Organisation (WHO) WellBeing Index* (WHO-5; WHO, 1998; Topp et al., 2015). A five item self-report measure of current mental wellbeing. Participants respond to statements such as, *I have felt cheerful and in good spirits* on a 5-point scale to indicate how they have felt over the previous two weeks whereby, 5 = All of the time = 5; Most of the time = 4; More than half of the time = 3; Less than half of the time = 2; Some of the time = 1. Responses are summed and then multiplied by 4 to give a final score, whereby 100 represents the best imaginable wellbeing (α = .88).

*The Big Five Inventory* (BFI; John et al., 1991; John et al., 2008). This 44-item measure provides a score for each of the Big Five personality traits. Participants indicate the extent to which each item describes them on a scale whereby 1 = completely disagree and 5 = completely agree to. We observed good reliability for all five subscales: Openness to Experience (e.g. *Is curious about many different things*, α = .75), Extraversion (e.g. *Generates a lot of enthusiasm*, α = .79). Neuroticism (e.g. *Worries a lot,* α = .85), Agreeableness (e.g. *Is helpful and unselfish with others*, α = .69) and Conscientiousness (e.g. *Does a thorough job*, α = .78).

### Time 2

Time 2 data were collected in August 2022. Since Time 1, there had been some changes to behavioural requirements in response to the emerging omicron variant with mask-wearing again compulsory in indoor public settings during December 2021 and January 2022. When Time 2 data were collected, masks were still recommended in crowded and enclosed spaces and when in contact with clinically vulnerable individuals, though they only remained compulsory in healthcare settings. However, advice to get tested if unwell remained and just over 40,000 positive cases were reported during the week commencing 6^th^ August 2022 when Time 2 data collection began (GOV.UK, 2022). Over 33 million UK residents had received their full dose of vaccination (2 shots plus a booster) by Time 2. Importantly in terms of public awareness, Covid-19 had virtually disappeared from the news headlines and media speculation about an end to the pandemic had begun (e.g. Katzourakis, 2022), even though the International Public Health Emergency declared in January 2020 remained (WHO, 2020).

#### Participants and procedures

The 183 participants whose data were included at Time 1 were contacted anonymously via Prolific and asked to take a further survey. This was again presented online via Qualtrics survey software. One hundred and twenty-five Time 1 participants responded (37 males, 87 females, 1 other; Mean age = 36.60, SD = 15.62). MacArthur Ladder Scale Mean SES rating was 5.36 (SD = 1.68, range 2–8). Fifty-five participants (42.6%) reported they or someone close to them had experienced Covid-19 infection since Time 1. Assuming Big Five personality traits to be stable over time, Time 2, we presented only the Silver Lining Questionnaire (SLQ; α = .96) and WHO-5 wellbeing scale (α = .85).

#### Analysis

Analyses were conducted using SPSS software, v.25. We used an intention-to-treat (ITT) method at Time 2 which allowed for inclusion of data for participants who dropped out after Time 1. Attrition rates are often higher in participants with certain characteristics (e.g. poor health), and an ITT approach reduces the risk of bias, while also preserving sample size. Also, as ITT results are generally conservative, the risk of a Type 1 error is minimised (Gupta, 2011). We calculated descriptive statistics at both timepoints and compared these using paired-samples t-tests. We then conducted two linear regression analyses. We removed the one participant who reported their gender as ‘other’ and included gender as a covariate (male = 0, female = 1), together with age, SES, and reports of Covid-19 infection (yes = 1, no = 0). Big Five trait scores were included alongside these covariates at Stage 1, and SLQ score was added at Stage 2. For the Time 2 data, we took a longitudinal approach, incorporating Time 1 wellbeing data as an additional factor at Stage 1 in order to examine change over time. We tested for mediating effects of SLQ score (our indicator of adversarial growth) on the relationships between personality traits and wellbeing, and wellbeing at Time 1 and wellbeing at Time 2. These analyses were conducted using the PROCESS macro for SPSS, v.3.5, employing model 4, which specifies simple mediation (Hayes, 2018). In our mediation analyses, all other variables were included as covariates as recommended by Hayes (2018). Finally, we examined the most highly reported silver linings at each timepoint, as indicated by endorsements to individual items on the SLQ.

## Results

Descriptive statistics are shown in Table 1. Participants reported an increase in wellbeing, *t =* 3.23, *df =* 182, *p* = .001, and in SLQ score, *t =* 2.89, *df =* 182, *p =* .01, between Times 1 and 2. At both timepoints, almost all participants answered in the affirmative to at least one SLQ item (Time 1 96%; Time 2 97%).

**Table 1.** Descriptive Statistics Across Both Timepoints.

	Time 1		Time 2	
	Mean	SD	Mean	SD
Wellbeing	54.17	21.43	57.92	20.00
SLQ score	15.08	9.85	16.08	10.11
Extraversion	3.09	.71	—	—
Agreeableness	3.86	.53	—	—
Conscientiousness	3.50	.67	—	—
Neuroticism	3.24	.84	—	—
Openness	3.73	.58	—	—

Table 2 presents the results of linear regressions on wellbeing. At Time 1, as expected, we observed a negative effect of neuroticism, and also a positive effect of openness to experience. At Stage 2 of the analysis these remained significant factors and SLQ score explained additional variance in wellbeing, as predicted, ΔR2 = .06; F (1, 171) = 16.21, *p* < .001.

**Table 2.** Results of Regression Analyses on Wellbeing.

		Time 1				Time 2			
				95% CI				95% CI	
		β	p	Lower	Upper	β	p	Lower	Upper
1	Age	−.005	.95	−.19	.18	−.05	.32	−.21	.07
	Sex	.07	.30	−2.72	8.85	−.05	.39	−6.32	2.47
	SES	.19	<.001	.87	4.49	−.04	.45	−1.94	.86
	Covid	−.03	.64	−7.55	4.68	−.02	.64	−5.82	3.58
	E	.11	.15	−1.23	7.72	.02	.72	−2.78	4.03
	A	.12	.10	−.94	10.38	.09	.12	−.86	7.78
	C	.03	.63	−3.42	5.60	.04	.54	−2.35	4.48
	N	−.34	<.001	−12.62	−4.65	−.17	.02	−7.08	−.73
	O	.21	<.001	2.91	12.40	.04	.49	−2.40	5.00
	WB Time 1	—	—	—	—	.61	<.001	.45	.68
	*Adj. R*^*2*^ *=* .30					*Adj. R*^*2*^ = .54			
2	Age	−.002	.99	−.18	.18	−.05	.35	−.20	.07
	Sex	.08	.22	−2.10	9.01	−.04	.45	−5.88	2.63
	SES	.20	<.001	1.05	4.52	−.02	.75	−1.59	1.15
	Covid	−.001	.99	−5.91	5.90	−.01	.79	−5.16	3.95
	E	.09	.20	−1.49	7.10	.01	.87	−3.02	3.57
	A	.05	.49	−3.64	7.54	.05	.41	−2.49	6.06
	C	.01	.92	−4.13	4.56	.01	.92	−3.16	3.51
	N	−.32	<.001	−11.98	−4.32	−.18	.01	−7.30	−1.15
	O	.16	.01	1.18	10.46	.00	.99	−3.67	3.62
	WB Time 1	—	—	—	—	.56	<.001	.40	.63
	SLQ score	.27	<.001	.30	.87	.21	<.001	.19	.63
	*Adj. R*^*2*^ *=* .36					*Adj. R*^*2*^ = .57			

Wellbeing reported at Time 1 presented a highly significant direct effect on Wellbeing at Time 2, with neuroticism also showing a significant negative direct effect. At Stage 2 of this regression, we entered SLQ score collected at Time 2. The effects of both Time 1 wellbeing and Neuroticism remained significant, with Time 2 SLQ score explaining additional variance, ΔR2 = .03, F (1, 170) = 13.05, *p* < .001.

**Figure 1.**
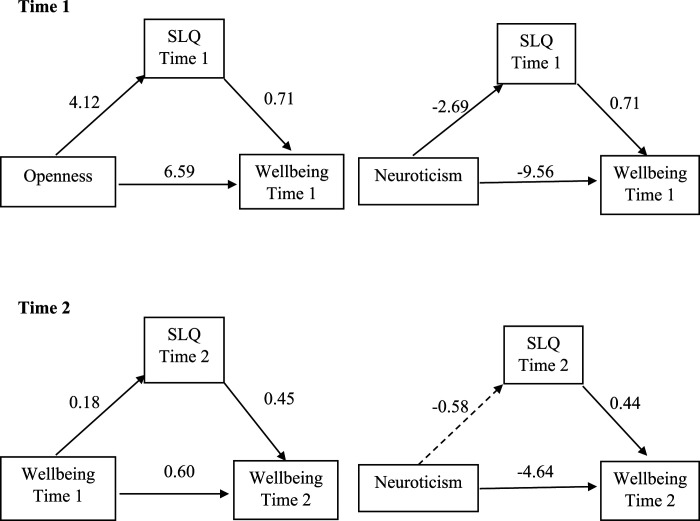
Mediation at each timepoint (solid lines indicate significant effects).

We tested the hypothesised mediating effects of SLQ score as presented in Figure 1.

At Time 1, SLQ score significantly positively mediated the relationship between Openness and wellbeing, *β =* 3.30, *95% CI* [1.09, 6.42], and between Neuroticism and wellbeing, *β =* −1.92, *95% CI* [–3.54, −.63]. At Time 2, SLQ score mediated the relationship between Wellbeing at Times 1 and 2, *β =* .08, *95% CI* [.04, .13], but not the relationship between neuroticism and wellbeing, *β =* −.25*, 95% CI* [–1.22, .52].

Finally, we examined responses to individual items in the SLQ to discover which were most highly endorsed (i.e. responses of agree or strongly agree) at each timepoint. As Table 3 shows, the silver linings for most participants were related to the strength and authenticity of relationships, with reflection or changed perspective on life more important at the later timepoint.

**Table 3.** The Five Most Highly Endorsed SLQ Items at Each Timepoint.

	Time 1		Time 2	
SLQ item	Rank	% endorsed	Rank	% endorsed
I Appreciate life more because of the pandemic.	1	68	1	75
The pandemic made me think about the true purpose of life.	2	62	3	66
The pandemic strengthened my relationship with others.	3	61		
The pandemic made me face up to problem areas of my life.	4	61	5	63
The pandemic has made me more mature.	5	58		
Because of the pandemic I find it easier to accept what life has in store.			2	72
The pandemic encouraged me to reflect on how I feel about myself.			4	64

## Discussion

The aim of this study was to examine the possibility of adversarial growth from the Covid-19 pandemic, the first to do so with a general public sample. We also aimed to identify individual differences associated with personality traits identified within the Big Five model. We examined the effects at two timepoints, nine months apart. As predicted, and in line with previous research in other contexts, adversarial growth (signified by scores on the silver-lining questionnaire, SLQ) was negatively associated with Neuroticism and positively with Openness to experience. However, we found no significant associations with other traits. We predicted that adversarial growth would account for variance in wellbeing over and above the effects of personality traits, and this prediction was upheld. Moreover, at Time 1, SLQ scores significantly mediated the effects of both Neuroticism and Openness to experience on wellbeing, again as predicted. Our results suggest that higher Openness increased the ability to perceive silver linings in the pandemic, thereby leading to greater wellbeing. Conversely, Neuroticism did not relate to perception of silver linings at Time 2. By Time 2, reported wellbeing had increased and regression analysis suggested that this was strongly associated with wellbeing levels at Time 1, in other words, those individuals who were happier at Time 1 were also likely to be happiest at Time 2. Importantly however, this association was mediated by SLQ scores, suggesting that wellbeing has increased most when individuals are also able to perceive silver linings. We observed a significant negative effect of neuroticism on wellbeing at Time 2, though no mediating effect.

That Neuroticism presented a consistent negative relationship with wellbeing was expected given the characteristics of the trait. Trait neuroticism is typified by low mood and poor coping, and is negatively associated with adversarial growth in general, and with wellbeing during Covid-19 specifically (Bacon et al., 2022). We also observed a positive effect of Openness to Experience on Wellbeing at Time 1. An open mind, and acceptance of change are important in adversarial growth (Linley & Joseph, 2004; Sheikh, 2004; Tedeschi et al., 2018; Tedeschi & Calhoun, 2004) and so it is unsurprising that a mediation effect of SLQ scores was also observed. We found no significant effects of other Big Five traits in the present study which is perhaps surprising given that Extraversion has been linked with adversarial growth in other contexts (Plews‑Ogan et al., 2019; Sheikh, 2004; Tedeschi & Calhoun, 2004; Val & Linley, 2006) and there is evidence that Agreeableness has supported adaptation and resilience ealier in the pandemic (Bacon et al., 2022). It may be that these traits are less important for wellbeing once the immediate Covid-19 threat had subsided and social restrictions removed.

It is notable that SLQ scores suggest that some level of adversarial growth was perceived by over 90% of participants in the present study, higher rates than previously reported. The review by Wu et al., (2019) suggested 53% of participants report growth follwing traumatic life events, and a recent study with nurses working through the pandemic reported 39% (Chen et al., 2020). Although the general public have experienced difficulties during the pandemic, they may be more likely to perceive silver linings than nurses who have been at the front line of healthcare, witnessed many cases of serious illness and death at first hand, and may be experiencing serious burnout and poor mental health (Galanis et al., 2021).

Our other prediction concerned the type of silver linings perceived. As expected, the most highly endorsed items on the SLQ were those related to a renewed appreciation of life, which was the top rated silver lining at both timepoints, and strenghtened relationships which was second most highly rated at Time 1. This has been a consistent finding in studies of adversarial growth in other domains, and social support; the perception that others care for you and will help you when needed, is a known correlate of resilience and of personal growth (Tedeschi & Calhoun, 2004; Wang et al., 2018). This may have been particularly important in view of the sense of isolation that social restrictions may have brought about during the pandemic. However, relationships did not not feature in the top five silver linings reported at Time 2. By this point, perceptions of a greater ability to cope with negative life events, together with self-reflection, were highly rated. This suggests a possible shift from an appreciation of relationships to an awareness of gains in personal development once social constraints were lifted and life returned to some semblance of normality. Living through a stressful event can lead to transformational positive coping, improved emotional functioning and thriving (O’Leary, et al., 1998). Indeed, growth in itself has been described as a reappraisal-based coping process whereby adaptation to adversity can lead to the development of positive psychological resources (Park & Folkman, 1998). Our data suggest this is indeed the case in Covid-19.

These results are of interest in terms of recent work suggesting that social identites were reinforced during Covid. Social identity theory explains how individuals seek to bolster self-esteem – and thereby wellbeing – amongst other ways, by turning towards their social (vs. individual) identities. The social connections arising from shared group membership and social identities have beneficial effects on health and wellbeing (Haslam et al., 2018; Jetten, et al., 2017) and, most recently, Carter et al. (2023) have demonstrated that identity-based social support has supported mental and physical health outcomes during COVID-19. Social connections may also lead to enhanced adversarial growth, espacially when an individual takes an active role, such as volunteering (Anderson et al., 2016). During the pandemic, a social movement campaign to clap-for-carers saw many UK neighbourhoods take to their doorsteps on a Thursday evening, with several million people estimated to take part (BBC News, 2020). Qualitative data has shown that this social action of expressing gratitude was a potent driver of positive affect, especially in the early parts of the lockdown (Day, et al., 2021). People who took part in similar collective acts of support in Spain reported enhanced sense of social connection, social identity and wellbeing (Canto & Vallejo-Martín, 2021). Fredrickson’s (2004) ‘broaden and build’ theory of gratitude suggests that positive emotions generate actions which in turn build durable resources that can be drawn on as coping strategies in times of adversity. Overall, actions such as helping someone out during the pandemic, or simply clapping one’s gratitude, together with an enhanced sense of connectedness, may have helped to generate adversarial growth. Nevertheless, an important question for further research is the longevity of the growth effect. Research with cancer patients (Danhauer et al., 2013) and war veterans (Tsai & Pietrzak, 2017) has suggested that adversarial growth is a dynamic process with varying trajectories depending on factors such as gratitude, purpose in life, spirituality, and social support. We can see how a greater sense of community identity may provide many of these benefits, but it remains to be seen whether that sense of identity will last. Further research might usefully examine this question and whether efforts to support the facilitating aspects of adversarial growth can maintain an ongoing sense of wellbeing.

As such, this research has implications for therapuetic intervention. That adversarial growth mediated the effects of personality traits on wellbeing, and appeared to support increase of wellbeing from Time 1 to Time 2, suggests that interventions which acknowledege changes to fundamental self-identity and which focus on supporting interpersonal relationships, appreciation of life, new possibilities and existential change may be effective. Tedeschi and Moore (2021) discuss an adversarial growth informed approach which integrates elements of cognitive-behavioral, narrative, interpersonal, and existential therapies while emphasising the therapeutic relationship in what they term expert companionship. They describe how the development of new life narratives, social support and belief systems, can lead ultimately to adversarial growth, wisdom, and the capability to be resilient to future trauma. In addtion, although the majority of our participants reported at least one silver lining, some did not. Interventions which encourage optimism, and the recognition of potential benefits of social support and revised life narratives might usefully bolster mental health in individuals who initially perceive little in the way of silver linings (Prati & Pietrantoni, 2009).

A clear strength of this research is the prospective design. We collected data from the same participants at two timepoints, rather than using retrospectively collected data as with many studies concerning Covid-19. This has allowed us to identify how the relationships between personality, wellbeing and adversarial growth, as well as the actual nature of perceived silver linings, may have changed over time, as society moved further from the immediacy of the pandemic. A further strength is that we employed participants from the general public, rather than specific populations with specialised experiences such as nurses, and our samples comprised participants with a range of social backgrounds and ages. Nevertheless, the study has limitations, including the reliance on self-report, and that our data do not allow for examination of the lasting effects of adversarial growth on individuals or communities. It is also important to note that we did not collect ethnicity details. There is evidence that people from some ethnic groups may experience more severe Covid-19 related health outcomes (Sze et al., 2020) and there may also be differences in how minority groups experienced changes in social identity. Future work might usefully focus more specifically on these populations.

In conclusion, despite the disruption and trauma which is reported to have arisen in the Covid-19 pandemic, many people have found positivity in an otherwise negative situation and their ability to do so has contributed to their wellbeing. The degree of adversarial growth partly depends on personality. We found evidence to suggest that openness particularly is positively, and neuroticism negatively, associated with adversarial growth. Importantly, adversarial growth can be experienced in different ways, and the particular form it takes may depend on the situation and timing in relation to negative experiences. Building on the ability to experience adversarial growth in the present and supporting individuals to identify opportunities to do so in future, may offer targets for intervention and help to assuage the anticipated psychological sequelae of this and future pandemics.

## Data Availability

The data that support the findings of this study are available from the corresponding author upon reasonable request.
